# Investigating shared aetiology between type 2 diabetes and major depressive disorder in a population based cohort

**DOI:** 10.1002/ajmg.b.32478

**Published:** 2016-08-02

**Authors:** Toni‐Kim Clarke, Jana Obsteter, Lynsey S. Hall, Caroline Hayward, Pippa A. Thomson, Blair H. Smith, Sandosh Padmanabhan, Lynne J. Hocking, Ian J. Deary, David J. Porteous, Andrew M. McIntosh

**Affiliations:** ^1^ Division of Psychiatry, University of EdinburghRoyal Edinburgh HospitalEdinburghUnited Kingdom; ^2^ Institute of Genetic MedicineNewcastle UniversityNewcastle upon TyneUnited Kingdom; ^3^ Centre for Genomics and Experimental Medicine, Institute of Genetics and Molecular MedicineUniversity of Edinburgh, Western General HospitalEdinburghUnited Kingdom; ^4^ MRC Human Genetics, MRC Institute of Genetics and Molecular MedicineUniversity of EdinburghEdinburghUnited Kingdom; ^5^ Centre for Cognitive Ageing and Cognitive EpidemiologyUniversity of EdinburghEdinburghUnited Kingdom; ^6^ Division of Population Health SciencesUniversity of DundeeDundeeUnited Kingdom; ^7^ Institute of Cardiovascular and Medical SciencesUniversity of GlasgowGlasgowUnited Kingdom; ^8^ Institute of Medical SciencesUniversity of AberdeenAberdeenUnited Kingdom; ^9^ Department of PsychologyUniversity of EdinburghEdinburghUnited Kingdom

**Keywords:** depression, type 2 diabetes, genetics, polygenic

## Abstract

Type II diabetes (T2D) and major depressive disorder (MDD) are often co‐morbid. The reasons for this co‐morbidity are unclear. Some studies have highlighted the importance of environmental factors and a causal relationship between T2D and MDD has also been postulated. In the present study we set out to investigate the shared aetiology between T2D and MDD using Mendelian randomization in a population based sample, Generation Scotland: the Scottish Family Health Study (N = 21,516). Eleven SNPs found to be associated with T2D were tested for association with MDD and psychological distress (General Health Questionnaire scores). We also assessed causality and genetic overlap between T2D and MDD using polygenic risk scores (PRS) assembled from the largest available GWAS summary statistics to date. No single T2D risk SNP was associated with MDD in the MR analyses and we did not find consistent evidence of genetic overlap between MDD and T2D in the PRS analyses. Linkage disequilibrium score regression analyses supported these findings as no genetic correlation was observed between T2D and MDD (rG = 0.0278 (S.E. 0.11), *P*‐value = 0.79). As suggested by previous studies, T2D and MDD covariance may be better explained by environmental factors. Future studies would benefit from analyses in larger cohorts where stratifying by sex and looking more closely at MDD cases demonstrating metabolic dysregulation is possible. © 2016 The Authors. *American Journal of Medical Genetics Part B: Neuropsychiatric Genetics* Published by Wiley Periodicals, Inc.

## INTRODUCTION

Major Depressive Disorder (MDD) is a complex psychiatric disorder characterized by persistent low mood, and is the second leading cause of disability worldwide [Ferrari et al., 2013]. The precise biological cause of MDD is unknown but significant overlap between MDD and somatic diseases has been noted [Goodwin, 2006]. Type II diabetes (T2D) has significant co‐morbidity with MDD and the odds of developing depression in T2D individuals are twice that of non‐type II diabetics [Anderson et al., 2001]. Bidirectional studies have found that the relative hazard for developing diabetes is 1.10 for each 5 unit increase in CES‐D scores (self‐reported depressive symptoms) and the relative hazard for MDD was 1.54 for untreated T2D [Golden et al., 2008]. The cause of this co‐morbidity is not fully understood. Shared environmental and genetic risk factors have been hypothesized to underlie MDD and T2D. Furthermore, a causal relationship may exist whereby the symptoms of diabetes cause depression in some individuals, and vice‐versa. By determining the underlying factors that promote the co‐occurrence of T2D and MDD we may be able to understand more about the biological basis of these traits.

Twin studies have traditionally been used to estimate the genetic contribution to the association between MDD and T2D. A study of male twins found no influence of genetic factors on the co‐expression of T2D and MDD [Scherrer et al., 2011]. Similarly, a study of Swedish twins found no effect of genetic factors, but that unique environmental factors significantly contribute to MDD and T2D [Mezuk et al., 2015]. A recent large study of Swedish and Danish Twin population registries found evidence of individual‐specific environmental factors in males whereas the correlations in females were due to genetic factors [Kan et al., 2016].

Large genome‐wide association studies (GWAS) of T2D and MDD have found that a substantial portion of genetic susceptibility is attributable to common genetic variants. A GWAS of T2D involving 38,840 cases and 114,981 controls found the proportion of genetic variance attributable to common genetic variants to be 49% on the liability scale [Morris et al., 2012]. Unlike MDD GWAS, which have only identified two genome‐wide significant loci to be associated with MDD in Chinese women [CONVERGE, 2015], GWAS of T2D have found 70 loci to be significantly associated with increased risk for T2D. These 70 loci account for 10.9% of the disease variance of T2D, a substantial portion of the 49% of variance explained by all common SNPs [Morris et al., 2012]. GWAS summary data can be used to test for genetic overlap between traits by creating polygenic risk scores (PRS). One study used 20 SNPs robustly associated with T2D and created an unweighted PRS however this was not significantly associated with depression in a large sample of ∼17,000 individuals [Samaan et al., 2015].

There may be a causal relationship between T2D and MDD. Individuals with depression may be more likely to have a poor diet [Sharma and Fulton, 2013], may exercise less or smoke; all of which increase risk for T2D. Conversely, MDD may arise from the distress caused by managing the symptoms of T2D. It is difficult to infer causality from observations alone as confounding factors such as socio‐demographics or education [Kessler and Bromet, 2013] may influence correlations.

Mendelian randomization (MR) is a technique that uses genetic factors as proxies for an environmental exposure of interest. MR assumes no pleiotropy; genetic factors should only be associated with the phenotype of interest via the environmental exposure. According to the laws of Mendelian inheritance regarding segregation and independent assortment, genetic variants will not be associated with confounding factors and therefore can help provide evidence of causal relationships [Smith and Ebrahim, 2003]. Individual SNPs can be weak instruments to investigate causality as they typically have small effects on phenotype expression and require large sample sizes to robustly detect associations. Polygenic risk scores (PRS), which aggregate the effect of thousands of SNPs into a score representing the overall burden of risk alleles an individual carries, have greater power to detect association between traits of interest. One limitation of using PRS to explore causal relationships is the risk of pleiotropy: associations may arise from causality or pleiotropy, particularly when thousands of SNPs comprise the PRS. Another technique, linkage disequilibrium (LD) score regression uses the LD information from SNPs to compute genetic correlations between traits of interest from GWAS summary statistics [Bulik‐Sullivan et al., 2015]. This method is typically better powered to detect genetic correlations compared to PRS and provides more reliable estimates of the magnitude of genetic overlap between traits.

The aim of this study was to investigate causal relationships and genetic overlap between T2D and MDD in the population based cohort, Generation Scotland: the Scottish Family Health Study (GS:SFHS) (N = 21,516) [Smith et al., 2006; Lee et al., 2013]. Using three techniques; MR, PRS, and LD score regression we aim to build evidence to explore the relationship between T2D and MDD with the hope of understanding more about the biological basis of these traits which will inform treatment of co‐morbid cases of T2D and MDD.

## MATERIALS AND METHODS

### Sample Description

#### Generation Scotland

The Scottish Family Health Study (GS:SFHS) is a family and population‐based study that recruited from the lists of General Practitioners throughout Scotland; the protocol for recruitment is described in detail elsewhere [Smith et al., 2006; Lee et al., 2013]. All components of GS:SFHS have received ethical approval from the NHS Tayside Committee on Medical Research Ethics (REC Reference Number: 05/S1401/89). Written consent for the use of data was obtained from all participants. GS:SFHS consists of 23,690 individuals over the age of 18 of whom 21,516 attended the research clinic. Genome‐wide genotype data were available for 19,858 individuals.

### Phenotype Definition

#### Depression and psychological distress phenotypes

The presence or absence of MDD was determined using the structured clinical interview for the Diagnostic and Statistical Manual of Mental Disorders (SCID) [First et al., 1997]. A brief screening questionnaire initially asked participants, “Have you ever seen anybody for emotional or psychiatric problems?” and “Was there ever a time when you, or someone else, thought you should see someone because of the way you were feeling or acting?” 21.7% of participants who answered yes to either of these questions went on to complete the SCID [First et al., 1997]. If they answered no to both of these questions, they were assigned control status. Individuals with a diagnosis of bipolar disorder were removed from this study. The General Health Questionnaire (GHQ‐28) was completed by 21,201 of participants providing a measure of current psychological distress [Goldberg and Hillier, 1979]. The GHQ‐28 consists of four subscales designed to assess: (A) somatic symptoms, (B) anxiety and insomnia, (C) social dysfunction and (D) “severe depression.” Total scores across subscales were used to provide a measure of current psychological distress using the GHQ scoring method. Scores were transformed towards normality using the BoxCox transformation procedure implemented in the MASS package in R. Continuous variables were scaled to have a mean of 0 and a standard deviation of one such that the reported beta‐coefficients are standardized.

#### Diabetes phenotype

Diabetes and medication use in GS:SFHS were self‐reported. The Scottish Diabetes Research Network (SDRN) provided information about T2D diagnoses which was linked to the Generation Scotland database [Anwar et al., 2011]. 915 individuals in GS:SFHS were assigned T2D status using self‐report data, SRDN diagnosis and sufficient medication information to distinguish between T1D and T2D (individuals using insulin were likely to be T1D). Individuals whose diabetes status (T1D vs T2D) was unclear or were confirmed as T1D using SDRN data were excluded from the analysis. Control individuals were those with no self‐reported diabetes, no evidence of diabetic medication use and no diagnosis from SDRN.

### Genotype Acquisition

Blood samples were obtained using standard operating procedures and were stored at the Wellcome Trust Clinical Research Facility Genetics Core (www.wtcrf.ed.ac.uk). Genotyping was carried out using the Illumina HumanOmniExpressExome‐8v1.0 BeadChip and Infinum chemistry24 and processed using the IlluminaGenomeStudio Analysis software v2011.1 (Illumina, San Diego, CA). Quality control removed SNPs with <98% call rate, SNPs with a Hardy–Weinberg *P*‐value ≤1 × 10^−6^ and a minor allele frequency greater than 1%. After quality control, 561,125 SNPs were available for analyses. The details of blood collection and DNA extraction are provided elsewhere [Smith et al., 2006].

### Mendelian Randomisation

The list of T2D risk SNPs selected for MR was made based on evidence for prior association with T2D. Single nucleotide polymorphisms (SNPs) found to be associated (*P* ≤ 5 × 10^−8^) with T2D in two GWAS (comprising 38,840 cases and 114,981 controls [Morris et al., 2012] and 47,979 cases and 139,611 controls comprising a trans‐ancestry GWAS [Mahajan et al., 2014]) were used to perform MR by testing for their association with MDD and current psychological distress. The list consisted of 10 independently associated SNPs from DIAGRAM GWAS that were significant at a genome‐wide level [Morris et al., 2012], and seven further independent loci identified in the DIAGRAM trans‐ancestry T2D GWAS [Mahajan et al., 2014]. 11/17 SNPs were directly genotyped in GS:SFHS and these were the SNPs used in this study (Table II). These SNPs have been validated for their association with T2D using a two‐stage meta‐analyses replication within the original GWAS studies. All SNPs have been found to be associated in European populations and are therefore suitable proxies for T2D in the present study. Using a Bonferroni correction for multiple testing we calculated the threshold for statistical significance for the MR analyses to be (*P* < 0.0045 [0.05/11]). PLINK was used to calculate the number of minor alleles to create a variable for association testing [Purcell et al., 2007]. Depression‐associated SNPs were not tested for association with T2D as the only two robustly associated MDD SNPs were identified in a sample of Chinese women, and do not replicate in the largest MDD GWAS of European descent [CONVERGE, 2015].

SNPs were tested for their association with MDD and GHQ‐28 scores in GS:SFHS using mixed linear models implemented in AS‐Reml‐R (www.vsni.co.uk/software/asreml) software package. Age, sex and SNP allele count were fixed effects. To control for relatedness between individuals family structure was fitted as a random effect by creating an inverse relationship matrix using pedigree kinship information. Wald's conditional F‐test was used to calculate the significance of fixed effects. If T2D SNPs are associated with MDD via a causal pathway involving diabetes then the association should only be present in diabetic individuals. A sensitivity analysis was carried out to determine these effects by testing for SNP association in diabetic and control individuals separately. As there were only 130 individuals in GS:SFHS with both diabetes and depression the sensitivity analysis was only performed for GHQ scores. If an association is observed in non‐diabetics then the SNPs may affect diabetes and depression independently (pleiotropy) and the assumptions of MR are violated.

### PRS Analysis

T2D and MDD PRS were computed for 19,858 genotyped individuals in GS:SFHS. T2D scores were created based on the DIAGRAM T2D GWAS summary data comprising 12,171 cases and 56,862 controls individuals [Morris et al., 2012] and MDD scores were computed based on the largest most recent MDD GWAS (N = 18,759) [Ripke et al., 2012]. Briefly, PRS were created in PLINK according to previously described protocols [Purcell et al., 2009]. Prior to creating scores, all strand‐ambiguous SNPs were removed from the GS:SFHS genotypes and SNPs were linkage disequilibrium pruned using clump‐based pruning (r2 = 0.25, 300 kb window). Five PRS were created for each trait using *P*‐value cut‐off thresholds of *P* ≤ 0.01, 0.05, 0.1, 0.5 and 1 for association in the original T2D and MDD GWAS. The association analyses of PRS with T2D/MDD were performed in AS‐REML‐R fitting family as a random effect as previously described. When T2D PRS was tested for association with GHQ or depression status, diabetes status was fit as a fixed effect covariate. Similarly, when MDD PRS was tested for association with diabetes status, depression status was fit as a fixed effect covariate. All models were controlled for age, sex, and four multidimensional scaling components to control for population stratification. The proportion of phenotypic variance explained by polygenic risk score was calculated by multiplying the profile score by its corresponding regression coefficient and estimating its variance. This value was then divided by the variance of the observed phenotype to yield a coefficient of determination between 0 and 1 [Nakawaga and Schielzeth, 2013]. Using a Bonferroni correction for multiple testing we calculated the threshold for statistical significance for the PRS analyses to be (*P* < 0.0017 [0.05/30]).

### LD Score Regression

GWAS summary statistics for the DIAGRAM T2D GWAS and the PGC MDD GWAS were used to perform LD score regression. This method uses the correlational nature of SNPs such that SNPs with high LD will have higher average χ^2^ statistics than those with low LD. To estimate genetic correlations the product of two z‐scores from GWAS of two traits can be regressed onto the LD score and the slope of the regression used to estimate genetic covariance [Bulik‐Sullivan et al., 2015]. The intercept was left unconstrained as the degree of sample overlap between DIAGRAM T2D and PGC MDD cohorts was unknown.

## RESULTS

Nine hundred and fifteen individuals in Generation Scotland were classed as Type II diabetics and 2,714 individuals met the criteria for a lifetime diagnosis of MDD. There was a significantly greater prevalence of MDD amongst T2D individuals in GS:SFHS (14.2% in T2D cases vs. 11.4% in T2D controls) and those with T2D had significantly higher GHQ scores (2.93 vs. 2.30) (Table I).

**Table I:** Comparison of Diabetic Individuals and Control Individuals for Measures of Depression and Psychological Distress in Generation Scotland

	Diabetic cases (N = 915)	Controls (N = 22,582)	*P*‐value
Age (S.E.)	59.4 (0.41)	47.3 (0.102)	6.05 × 10^−125^
Female	468 (51.2%)	13,412 (59.3%)	2.2 × 10^−10^
MDD	130 (14.2%)	2567 (11.4%)	2.6 × 10^−6^
MDD episode count	12.2 (1.49)	7.7 (0.27)	0.00012
GHQ total (S.E.)	2.93 (0.175)	2.3 (0.028)	7.9 × 10^−8^
MDD age of onset (S.E.)	36.6 (1.13)	31.2 (0.26)	0.74

Of the 11 SNPs previously identified as demonstrating association with T2D, only one was nominally associated with MDD in GS:SFHS. The A allele of rs6808574 was found to be negatively associated with MDD in GS:SFHS (beta = −0.008, *P* = 0.02) (Table II). This is the same allele found to be associated with decreased risk for T2D in the trans‐ancestry GWAS of T2D. No other SNPs were found to be associated with MDD in the MR analysis. Only one SNP was nominally associated with GHQ scores, rs3130501. The A allele of this SNP was associated with lower GHQ scores (beta = −0.025, *P*‐value = 0.03) (Table III) in GS:SFHS and with decreased risk for T2D in the trans‐ancestry GWAS of T2D. Further analyses of rs3130501 and GHQ scores show it was associated with GHQ score in non‐diabetic controls (beta = −0.03, *P*‐value = 0.01). However, although diabetic cases showed a stronger correlation with GHQ score (beta = −0.04) this association was not significant (*P* = 0.56) as only 915 diabetic cases were available for analysis in GS:SFHS (Table III). This sensitivity analysis suggests that any relationship between this SNP and GHQ arises via pleiotropic effects rather than a causal relationship between diabetes and psychological distress. None of the associations between T2D SNPs and MDD or GHQ remained significant after correction for multiple testing. Thus, MR analysis provided no evidence for a causal relationship between T2D and MDD.

**Table II:** Mendelian Randomisation Analyses of T2D associated SNPs With MDD Status in GS Controlling for Age and Sex

SNP	Beta (S.E.)	Z‐ratio	*P*‐value
rs10401969	0.007 (0.007)	0.97	0.33
rs10842994	0.003 (0.005)	0.57	0.57
rs12970134	0.003 (0.004)	0.79	0.43
rs2796441	0.0009 (0.004)	0.25	0.80
rs3130501	0.007 (0.004)	1.68	0.09
rs4275659	−0.001 (0.004)	−0.26	0.79
rs516946	−0.003 (0.004)	−0.50	0.56
rs6808574	−0.008 (0.003)	−2.29	**0.02**
rs702634	0.001 (0.004)	0.33	0.74
rs7177055	−0.003 (0.004)	−0.66	0.51
rs7202877	0.0009 (0.006)	0.15	0.88

Nominally significant SNPs are highlighted in bold.

**Table III:** Association of T2D Associated SNPs With GHQ Total Scores in GS Full Cohort, Diabetics Only and Controls Only

Full Sample				Diabetics only			Controls only		
SNP	Beta (S.E.)	Z‐ratio	*P*‐value	Beta (S.E.)	Z‐ratio	*P*‐value	Beta (S.E.)	Z‐ratio	*P*‐value
rs10401969	0.014 (0.02)	0.66	0.51	0.10 (0.1)	1.03	0.30	0.009 (0.02)	0.44	0.66
rs10842994	−0.0019 (0.01)	−0.15	0.88	0.002 (0.07)	0.03	0.98	−0.004 (0.01)	−0.31	0.75
rs12970134	0.013 (0.01)	1.12	0.26	0.07 (0.06)	1.19	0.24	0.013 (0.01)	1.08	0.27
rs2796441	0.016 (0.01)	1.55	0.12	0.07 (0.05)	1.30	0.20	0.013 (0.01)	1.29	0.20
rs3130501	−0.025 (0.01)	**−2.17**	**0.03**	**−0.04(0.07)**	**−0.59**	**0.56**	**−0.03 (0.01)**	**−2.56**	**0.01**
rs4275659	−0.018 (0.01)	−1.62	0.11	−0.053 (0.06)	−0.84	0.40	−0.02 (0.01)	−1.65	0.10
rs516946	−0.017 (0.01)	−1.37	0.17	0.054 (0.07)	0.81	0.42	−0.02 (0.01)	−1.85	0.06
rs6808574	0.014 (0.01)	1.31	0.19	−0.014 (0.06)	−0.24	0.81	0.02 (0.01)	1.39	0.16
rs702634	−0.009 (0.01)	−0.78	0.43	0.001 (0.06)	0.02	0.98	−0.006 (0.01)	−0.56	0.57
rs7177055	−0.009 (0.01)	−0.79	0.43	−0.12 (0.06)	−2.09	0.04	−0.005 (0.01)	−0.43	0.66
rs7202877	−0.014 (0.02)	−0.82	0.41	−0.03 (0.10)	−0.29	0.77	−0.01 (0.02)	−0.60	0.55

Controlled for age and sex. Nominally significant SNPs highlighted in bold.

PRS analyses found the T2D PRS to be associated with T2D in GS:SFHS at 5 out of 5 *P*‐value thresholds with the *P* ≤ 0.05 threshold explaining most of the variance in T2D status (beta = 0.013, r^2^ = 0.004, *P*‐value = 1 × 10^−18^), indicating that the T2D PRS is a valid instrument for use in GS:SFHS. MDD PRS was associated with MDD at 4 out of 5 *P*‐value thresholds in GS:SFHS with the *P*‐value threshold explaining most of the variance being *P* ≤ 1 (beta = 0.011, r^2^ = 0.003, *P*‐value = 3 × 10^−5^). Similarly, this MDD PRS explained most of the variance in GHQ scores in GS:SFHS (beta = 0.046, r^2^ = 0.053, *P*‐value = 6 × 10^−10^) (Table IV). Cross association analyses found the T2D PRS to be nominally associated with MDD status at 3 out of 5 *P*‐value thresholds, the most strongly associated at *P* ≤ 1 (beta = 0.007, r^2^ = 0.001, p‐value = 0.015), after controlling for diabetes status, however this was not significant after correction for multiple testing. No association between T2D PRS and GHQ scores were found. MDD PRS were not significantly associated with T2D status in GS:SFHS (Table V).

**Table IV:** Validation of PGRS: Association of T2D PGRS With Diabetes Status and Association of MDD PGRS With Depression and GHQ Score in Generation Scotland

	T2D PGRS association with GS diabetes status			MDD PGRS association with GS depression status			MDD PGRS association with GS GHQ score		
*P*‐value threshold	Beta (S.E.)	*P*‐value	r^2^	Beta (S.E.)	*P*‐value	r^2^	Beta (S.E.)	*P*‐value	r^2^
0.01	0.001 (0.001)	3.83 × 10^−12^	0.0026	0.004 (0.003)	0.102	0.0005	0.036 (0.008)	1.81 × 10^−6^	0.029
0.05	0.013 (0.001)	1.06 × 10^−18^	0.0042	0.008 (0.003)	0.002	0.0017	0.043 (0.007)	6.61 × 10^−9^	0.046
0.1	0.012 (0.001)	1.21 × 10^−15^	0.0035	0.010 (0.003)	0.0001	0.0026	0.043 (0.007)	8.34 × 10^−9^	0.046
0.5	0.012 (0.001)	5.11 × 10^−16^	0.0036	0.010 (0.003)	0.0001	0.0026	0.046 (0.007)	6.38 × 10^−10^	0.053
1	0.012 (0.001)	1.07 × 10^−15^	0.0036	0.011 (0.003)	0.00003	0.0030	0.046 (0.007)	5.75 × 10^−10^	0.053

Controlling for age, sex, and 4 MDS components.

**Table V:** Cross Association PGRS Analyses: Association of T2D PGRS With Depression and GHQ Score, Adjusting for Diabetes Status, Age, Sex, and 4 MDS Components

	T2D PGRS association with depression			T2D PGRS association with GHQ			MDD PGRS association with diabetes		
*P*‐value threshold	Beta (S.E.)	*P*‐value	r^2^	Beta (S.E.)	*P*‐value	r^2^	Beta (S.E.)	*P*‐value	r^2^
0.01	−0.0013 (0.003)	0.625	0.00004	−0.0081 (0.008)	0.287	0.002	0.0013 (0.001)	0.359	0.00004
0.05	0.0025 (0.003)	0.359	0.00016	0.0014 (0.008)	0.861	0.0001	0.0001 (0.001)	0.471	0.00003
0.1	0.0052 (0.003)	**0.048**	0.00073	−0.00043 (0.008)	0.956	0.00003	0.0003 (0.001)	0.850	0.000002
0.5	0.0061 (0.003)	**0.026**	0.00099	−0.0025 (0.008)	0.749	0.00002	0.0005 (0.001)	0.710	0.000007
1	0.0065 (0.003)	**0.015**	0.00112	−0.0025 (0.008)	0.748	0.00008	0.018 (0.001)	0.815	0.000003

Association of MDD PGRS with diabetes status, adjusting for depression status, age, sex, and 4 MDS components.

Nominally significant associations are highlighted in bold.

LD score regression using DIAGRAM T2D and PGC‐MDD GWAS summary statistics found no evidence to suggest shared genetic effects between T2D and MDD (Genetic Correlation (rG) = 0.0278 (S.E. 0.11), *P*‐value = 0.79).

## DISCUSSION

Using genetic factors to analyse the relationship between T2D and MDD we find little evidence that T2D is causally related to depression or psychological distress among GS:SFHS individuals. One SNP, rs6808574, was nominally associated with MDD and another, rs3130501, with GHQ. However, the association between rs3130501 and GHQ scores was found in non‐diabetic individuals indicating that the association arises from genetic pleiotropy rather than a causal relationship between T2D and psychological distress as these individuals are not self‐reporting T2D or registered in the SDRN as being diabetic. As nine out of eleven T2D‐associated SNPs failed to show any association with MDD or GHQ scores it would suggest that T2D is not causally related to depression or psychological distress. There was little evidence of genetic association between MDD and T2D when we applied a PRS analysis. T2D PRS showed some nominal association with MDD status at the less stringent inclusion thresholds of *P* ≥ 0.1. The MDD PRS was not associated with T2D status in GS:SFHS at any of the five p‐value thresholds. LD score regression found no evidence of genetic overlap between T2D and MDD using DIAGRAM and PGC summary statistics. These findings suggest there is little genetic overlap between T2D and MDD.

Our results are partially supported by other studies utilizing twin registries to examine the genetic contribution to MDD and T2D covariance. A study of Swedish twins found that non‐shared environmental factors are responsible for the majority of the association between T2D and MDD [Mezuk et al., 2015]. Another study of Swedish and Danish twins found a genetic contribution to the covariance in T2D and MDD amongst females in the Swedish sample whereas unique environmental effects were more influential in male twins. Genetic effects were contributing to T2D and MDD in males and females separately in the Danish sample, however, they found differences in the genetic effects between males and females in both samples, suggesting that future studies may benefit from stratifying by sex [Kan et al., 2016]. Similar to our study, a PRS analysis in a large sample including >3000 MDD cases found no association between a T2D PRS comprising 20 SNPs and MDD [Samaan et al., 2015].

We were not able to find consistent evidence for genetic overlap between T2D and MDD or evidence of a causal relationship leading from T2D to MDD. There are a number of limitations to our study which may have reduced our ability to detect an association between T2D and MDD. We were constrained by the number of diabetic individuals in GS:SFHS, only 915. Furthermore, we had to distinguish between T1D and T2D based on medication data and links to the SRDN database. Individuals with an ambiguous T1D/T2D status were removed but it is remains a possibility that there is some clinical heterogeneity unaccounted for in our sample. The SNPs used in the MR analyses had, individually, a small effect on risk for T2D in the original GWAS (OR = 1.06–1.13). Such small effects require large sample sizes to detect association and therefore a sample with more MDD cases should be used to test for a causal relationship between T2D and MDD in future studies.

Another limitation was the sensitivity of the MDD PRS compared to the T2D PRS due to the number of individuals in the original GWAS (MDD N = 18,759 vs. T2D N = 149,821). With a larger MDD GWAS and more T2D cases we may have uncovered a genetic overlap using PRS. We did find a nominal association between T2D PRS and MDD at higher p‐value thresholds and therefore this should be investigated further using a larger sample. However, LD score regression using the same GWAS summary statistics use to create PRS in this study found no genetic overlap between T2D and MDD. Another limitation is that although we used medication data and linkage to the SDRN to assign T2D case status, for some individuals self‐report was the only measure available and this may have led to some individuals being misclassified.

A recent study of depressive symptoms and T2D risk in 2525 Canadian individuals found that the risk for T2D was only increased in those reporting depressive symptoms and presenting with metabolic dysregulation, characterized by obesity, high blood pressure, elevated blood sugar and high triglycerides [Schmitz et al., 2016]. It may be that there is a sub‐type of MDD characterized by metabolic dysregulation which has genetic overlap with T2D. Future studies of larger cohorts may benefit from stratifying depression according to metabolic profile and looking at the sources of covariance with T2D. Future MR studies would also benefit from investigating the association between MDD associated SNPs and T2D. We were unable to study this, as no robustly associated genome‐wide significant SNPs are associated with MDD in individuals of European ancestry. As sample sizes for MDD GWAS become larger and more loci are identified these analyses can be carried out to determine whether a causal relationship leading from MDD to T2D exists. Future studies of larger cohorts would benefit from stratifying by sex and by MDD subtypes such as metabolic dysregulation to understand the co‐morbidity between T2D and MDD.

We conclude that there is little evidence for genetic overlap between T2D and MDD or a causal relationship leading from T2D to MDD. As suggested by other studies, the co‐expression of T2D and MDD is likely to be influenced by unique environmental factors.
